# Advanced primary vaginal squamous cell carcinoma: A case report and literature review

**DOI:** 10.3389/fimmu.2022.1007462

**Published:** 2022-11-22

**Authors:** Yue Shen, Xiangkai Meng, Lili Wang, Xiaoxiao Wang, Hua Chang

**Affiliations:** Department Gynecology, The First Hospital of China Medical University, Shenyang, Liaoning, China

**Keywords:** vaginal squamous cell carcinoma, chemotherapy, radiotherapy, immune checkpoint inhibitors, tyrosine kinase inhibitors, case report

## Abstract

**Background:**

Vaginal carcinoma is a gynecological malignancy with low incidence, and there are few relevant and specific guidelines for vaginal cancer in our country and abroad. Here, we report the case who was diagnosed with advanced, primary vaginal squamous cell carcinoma and underwent integrated treatment successfully.

**Case introduction:**

A 64-year-old Chinese woman underwent subtotal hysterectomy for uterine fibroids in 1998 and laparoscopic extensive residual cervical resection, bilateral ovarian salpingectomy, and pelvic lymph node dissection for residual cervical adenocarcinoma (stage IB1) in the First Affiliated Hospital of China Medical University in 2018. There was no postoperative review. The patient experienced vaginal discharge in March 2020, and vaginal bleeding occurred in July 2020. Our patient was diagnosed with stage IVA vaginal squamous cell carcinoma, based on a gynecological examination, colposcopy biopsy with histopathological examination, computed tomography scan, and tumor marker levels by two professors. After three phases of treatment (sequential treatment with chemotherapy plus radiotherapy, chemotherapy combined with immune checkpoint inhibitors, and immune checkpoint inhibitors combined with tyrosine kinase inhibitors therapy), her condition improved. Her current state is generally good, and she has achieved complete remission.

**Conclusion:**

We report a rare case of a patient with primary advanced vaginal carcinoma combined with cervical adenocarcinoma. The patient was treated for approximately 2 years, and her personalized treatment showed promising results. We will continue to follow up with the patient and monitor her response to the current treatment process.

## 1 Background

Vaginal carcinoma is a gynecological malignancy with low incidence and frequently occurs in postmenopausal women. It occurs in 0.9 per 100,000 women and causes 0.2 deaths per 100,000 women (1). The most common histological type is squamous cell carcinoma (80%), followed by adenocarcinoma (15%), with the rare cases of melanoma, lymphoma, and sarcoma accounting for the remaining 5% (2). Owing to the difficulties associated with conducting large, prospective, randomized trials in these rare tumor populations, all treatment options recommended by current guidelines can be applied to cervical malignancy. Simultaneously, owing to its special anatomical location, it can seriously affect the physical health, mental health, and quality of life of patients, although the mortality rate associated with early lesions is low. Here, we report the case of a patient with advanced, primary vaginal squamous cell carcinoma who successfully underwent integrated treatment for 22 months, along with a review of the relevant literature.

## 2 Case presentation

**Figure 1:**
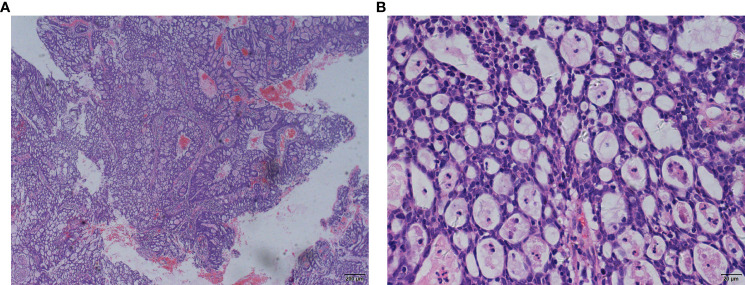
**(A, B)** Biopsy pathology. (August 7, 2020).

A 64-year-old woman underwent subtotal hysterectomy in 1998 for uterine fibroids. In 2018, she visited a doctor for postmenopausal vaginal bleeding for 3 months, and her physical examination revealed that the residual cervix had vegetation. A biopsy with histopathological examination suggested adenocarcinoma with high-grade squamous intraepithelial lesions (**Figures 1A, B**). Later, laparoscopic extensive residual corpectomy, bilateral ovarian salpingectomy, and pelvic lymph node dissection were performed. After the postoperative pathological diagnosis of residual terminal cervical adenocarcinoma stage IB1 (FIGO2009 stage), there was no postoperative review.

**Figure 2:**
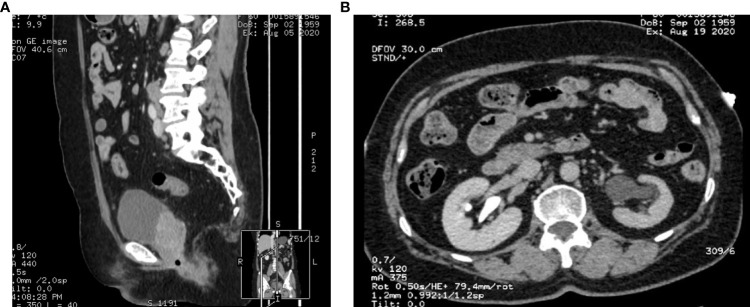
**(A, B)** Abdominal computed tomography (August 5, 2020).

Intermittent vaginal flow occurred in March 2020. Her vaginal secretions were yellow and had a peculiar smell. In July 2020, she experienced vaginal bleeding with pale pink blood. During physical examination, lesions of stump vagina was crunchy and contact bleeding were noted, with a maximum diameter of approximately 5 cm. She was positive for HPV16 and had a squamous cell carcinoma (SCC) antigen level of 22.50 ng/mL. At the outpatient clinic, simultaneous lesion biopsy with histopathological examination revealed SCC (moderate differentiation). Whole-abdominal contrast-enhanced computed tomography (CT) revealed a soft tissue mass on the upper left side of the vaginal stump with a maximum size of 4 cm × 3 cm, left renal effusion, atrophy, and multiple enlarged lymph nodes in the retroperitoneal area, indicating metastasis (**Figures 2A, B**). Cystoscopy revealed a mucosal bulge in the region of the left ureteral orifice covering approximately 3 cm × 2.5 cm, extending to the left posterior wall and left triangular area, with necrosis in the central region and peripheral follicular changes, easy bleeding, and no left ureteral orifice is seen. Based on these findings, she was diagnosed with residual vaginal SCC stage I VA (bladder, left ureter).

The patient underwent a left radical mastectomy for breast cancer in 2009, followed by postoperative chemoradiotherapy and tamoxifen endocrine therapy without detailed medical records. She had been pregnant three times, delivered one time, and miscarried two times without family history of cancer, smoking or drinking. She did not agree to undergo genetic testing due to financial problems.

### 2.1 Sequential treatment with chemotherapy plus radiotherapy

#### 2.1.1 Chemotherapy plus radiotherapy

**Figure 3:**
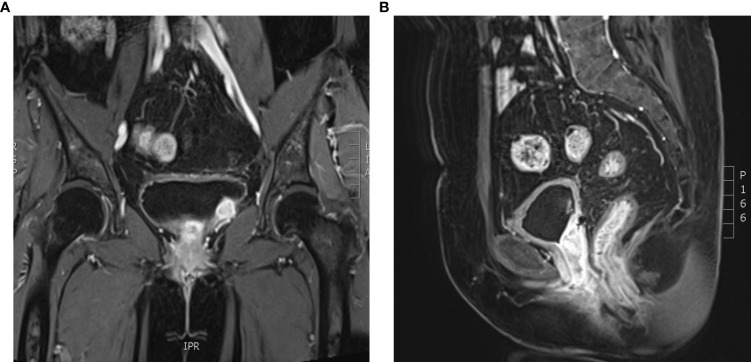
**(A, B)** Pelvic magnetic resonance imaging (September 23, 2020).

**Figure 4:**
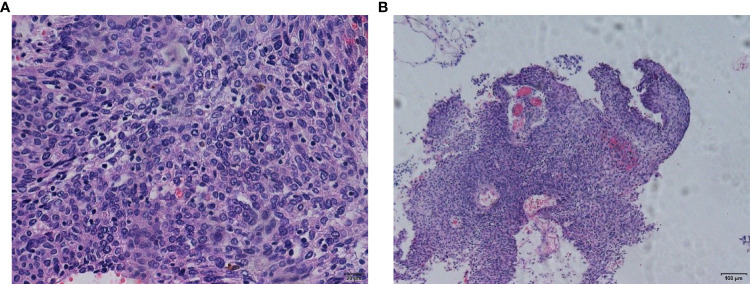
**(A, B)** Colposcopy and biopsy pathology (January 27, 2021).

Left nephrostomy was performed to relieve her symptoms from August 20, 2020, to September 11, 2020. We performed 28 external procedures in the radiotherapy department of our hospital from September 21, 2020, to October 31, 2020 (50.4 Gy/28f) for vaginal dissection lesions. On evaluation after radiotherapy, partial remission was noted (**Figures 3A, B**). The renal findings were as follows: TGFR, 56.4 mL/min; LGFR, 9.1 mL/min; and RGFR, 47.3 mL/min. Owing to the high renal toxicity of cisplatin, therapy involving the substitute docetaxel combined with carboplatin (intravenous chemotherapy) was completed according to the patient’ ‘s condition. After three sessions of chemotherapy, a 1*1cm nodule with a brittle texture was seen in the middle 1/3 of the anterior vaginal wall, and biopsy pathology suggested squamous carcinoma (**Figures 4A, B**). Vaginal brachytherapy was performed 6 times (5 Gy/time) in the radiotherapy department. Total abdominal CT after radiotherapy revealed a nodule in the posterior bladder wall with a large diameter of approximately 1.8 cm, which was evaluated as partial remission.

#### 2.1.2 Chemotherapy, radiotherapy and immune checkpoint inhibitors

Combined with patient biopsy pathological CPS≈10, therapy involving docetaxel plus carboplatin plus sintilimab was administered for two courses (the interval between the two courses was 42 days due to the COVID-19 pandemic). The CT images revealed that hypoechoic regions were visible in the posterior bladder wall and the left posterior bladder wall, measuring approximately 2.68 cm × 1.79 cm and 2.78 cm × 2.12 cm, respectively. Extra-pelvic irradiation was performed 20 times in the radiotherapy department (clinical target volume, 2 Gy/time [3 times] + gross tumor volume, 2 Gy/time [17 times]) for lesions in the bladder. Magnetic resonance imaging after radiotherapy suggested that the left paramural size of the urinary bladder was approximately 1.3 cm × 1.4 cm, the lesion and bladder were unclear, and the lateral lesion size of the vaginal stump was approximately 1.8 cm × 2.8 cm.

**Figure 5:**
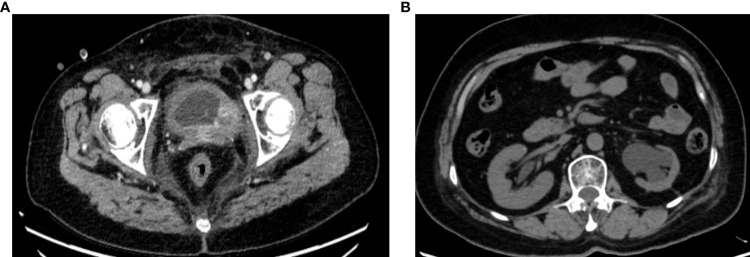
**(A, B)** Urinary tract computed tomography urography (October 24, 2021).

Ultrasonography after the second course of sintilimab indicated that the bladder was slightly filled, the wall was not smooth, and the left side of the bottom wall was thickened and irregular. The thickness was approximately 1.08 cm. Hyperechogenicity was noted in the vagina with a size of approximately 7.15 cm × 4.30 cm × 4.42 cm (**Figures 5A, B**).

### 2.2 Chemotherapy combined with immune checkpoint inhibitors

**Figure 6:**
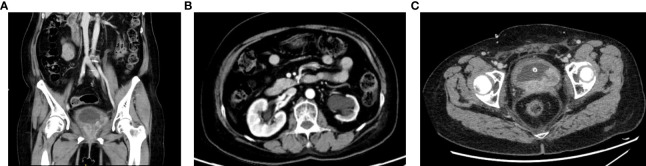
**(A–C)** Abdominal computed tomography (January 6, 2022).

Due to the disease progression, the treatment regimen was changed to paclitaxel for injection (albumin bound) plus sintilimab for six cycles. A review of the left posterior wall of the bladder revealed a region with slightly lower echogenicity, measuring approximately 4.17 cm × 3.20 cm × 4.42 cm (**Figures 6A–C**). A urethral region behind the vagina showed visible hypoechogenicity with a size of approximately 2.99 cm × 1.30 cm × 2.55 cm. Moreover, the SCC antigen level reduced from 8.85 ng/mL to 3.76 ng/mL.

### 2.3 Immune checkpoint inhibitors combined with tyrosine kinase inhibitors therapy

**Figure 7:**
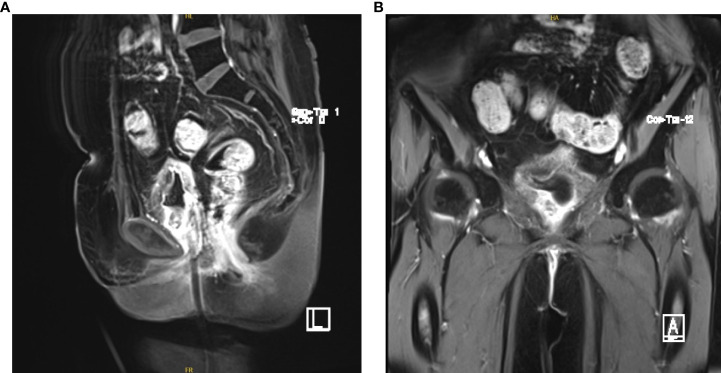
**(A, B)** Pelvic magnetic resonance imaging (May 17, 2022).

Studies have shown that anlotinib combined with sintilimab exhibits better efficacy and acceptable toxic side effects in patients with persistent, recurrent, or metastatic cervical cancer (3). In search of better efficacy, the patient switched to anlotinib plus sintilimab in March 2022. At present, five courses of this regimen have been administered, and complete response has been achieved (**Figures 7A, B**) (HPV negative; SCC antigen level, 0.81 ng/mL). No swelling was observed on examination of the vaginal end, and the pelvic cavity was empty. Ultrasonography suggested the thickening of the anterior and posterior walls of the bladder, with uneven echoes.

## 3 Discussion

Primary vaginal cancer accounts for 10% of all vaginal malignancies (4) and only 1%–2% of all gynecological cancers (5). The diagnosis of primary vaginal cancer does not include any cervical and/or vulvar lesions occurring within five years of cervical cancer treatment and any malignant lesions occurring within the vagina (1). Tumors in the vagina that extend to or involve the cervix are categorized as cervical cancer (6). The incidence of vaginal cancer increases with age and is most commonly seen in elderly and perimenopausal women. The incidence of primary vaginal cancer is increasing in young women due to increased persistent human papillomavirus(HPV) infection. Vaginal cancer is closely associated with HPV infection, and its risk factors include HSIL, smoking and immunosuppression (7–9). Patients with a history of cervical cancer are at increased risk of developing vaginal cancer because these sites are associated with HPV-associated infections (9).

Among histological type, the most commonly subtype is squamous cell carcinoma (SCC), which accounts for 90% of cases of vaginal carcinoma (6). Adenocarcinoma accounts for 8% to 10%, with the highest incidence among 17 to 21-year-olds. The incidence of vaginal clear cell adenocarcinoma is very low and most common in patients under 30 years of age, which is associated with adenopathy and exposure to hexestrol (DES) in uterine (10). Vaginal melanoma, sarcoma, and lymphoma are extremely rare.

Most vaginal cancers occur at the tip of the vagina, and lesions involving the upper part of the posterior wall often metastasize easily to the pelvic lymph nodes, including the obturator, internal iliac, and external iliac lymph nodes, whereas distal vaginal lesions metastasize easily to the inguinal and femoral lymph nodes. Lesions in the middle of the vagina may follow the pelvic or inguinal lymph node pathways (5). Vaginal carcinoma can spread directly to the paracervical tissues, vulva, and near the bladder and rectum, in addition to the lymphatic spread. Besides, vaginal carcinoma spreads to lung, liver, and bone by blood in the advanced stages (10). Thus, the metastatic route varies with the site and extent of the primary tumor.

Staging of vaginal cancer currently follows FIGO staging (11). In this case, although the diagnosis was stage IB1 cervical adenocarcinoma in 2018, it was jointly diagnosed as stage IVA (bladder and lower left ureter) based on the patient’ ‘s history, gynecological examination, imaging examination, and biopsy with histopathological examination.

The most important prognostic factor in vaginal carcinoma is the stage at diagnosis. In a large series of patients with vaginal cancer, the 5-year relative survival rates for stages 0, I, II, III, and IV were 96%, 64%–84%, 53%–58%, 36%, and 18%–36%, respectively. Other risk factors for a poor disease prognosis include a tumor size of >4 cm, advanced age, and the possibility that the tumor is located outside the upper third of the vagina (12–14). Histological subtypes also affect prognosis, with adenocarcinoma having a worse prognosis than SCC (6). In contrast, high-risk HPV DNA, low KI-67/MIB-1 expression, and P16+ were associated with favorable prognostic value.

The common signs of vaginal cancer includes painless vaginal bleeding and vaginal discharge, as well as postcoital or postmenopausal bleeding (10, 15). It can invase or compress nearby organs, resulting in urinary diseases such as urinary retention, dysuria, hematuria, or gastrointestinal symptoms such as tenesmus, constipation, or melena. Locally advanced disease is also associated with pelvic pain (15). In this case, we wanted to try to relieve the symptoms by ureteral stent tube placement because the lesion involved the bladder and the left ureteral opening, and hematuria and dyspareunia were present. Still, unfortunately, the cancerous lesion completely blocked the left ureteral orifice. Percutaneous left nephrostomy was performed to relieve left hydronephrosis during treatment, and the fistula was closed when the disease was controlled, and there is no hydronephrosis or other conditions at present.

Magnetic resonance imaging (MRI) provides better soft tissue contrast than computed tomography (CT) and is more sensitive in detecting local tumors, including size, paragvaginal and pelvic wall invasement rates. Vaginal cancer shows moderate to high signal intensity on T2-weighted MRI, and vaginal gel separation of the vaginal wall helps to visualize the tumor (6) better. PET-CT was used to evaluate lymph node metastasis and distant metastasis.

Vaginal cancer is the only cancer of the female reproductive system that has no clinical practice guidelines in the National Comprehensive Cancer Network, and standard treatment has not yet been established for this condition. Common multiway therapies include surgery, radiotherapy, and/or chemotherapy. Due to the rarity of vaginal cancer, there are no randomized controlled trials which can guide treatment decisions, and clinical guidelines are based on limited retrospective and comparative studies (10, 14). As a result, there is no standardized approach to managing and treating vaginal cancer, and a wide variety of treatment strategies are recognized for the condition.

The commonly used multimodality of treatment includes surgery, radiation, and/or chemotherapy (16). Surgery plays a limited role in the treatment of vaginal cancer, and the international federation of gynecology and obstetrics recommends that primary surgery should only be performed for patients with early disease and small lesions that are limited to the mucosa (17). Total pelvic clearance can play a role in patients with stage IV vaginal cancer with a rectovaginal or vesicovaginal fistula or patients with central recurrence after radiotherapy (6).

Radiotherapy provides treatment choices for most patients with vaginal cancer, particularly those with advanced forms of the disease. It involves a combination of external irradiation therapy and brachytherapy. A review showed that EBRT plus brachytherapy provide improved outcomes as compared toEBRT alone for advanced cancers stage III-IVA (16). The advantage of radiotherapy (18) is that it can preserve the vagina and other organs. Since the high recurrence rate, brachytherapy alone is not recommended for most tumors, even early-stage tumors. An extensive national cancer database (NCDB) study showed that CCRT was an independent prognostic factor for better overall survival (56 months for CCRT and 41 months for radiotherapy). The most commonly used regimen was cisplatin 40mg/m2/week.

Wanted to preserve fertility and sexual function of young patients with vaginal cancer also occur and these cases, the preferred treatment, patients with stage IV and distant recurrence in patients with metastasis, also needs systemic therapy, the effective scheme for systemic treatment of vaginal cancer, there is no consensus, these data may be inferred from the cervical cancer data. These data include immunotherapy and other targeted agents that are still under development.

For the treatment process of this patient, we think there are several highlights. (1) After the first diagnosis, sequential several highlights treatment was preferred because the lesion invaded the ureteral orifice and caused left hydronephrosis. For the patient’s left hydronephrosis, we tried to relieve the symptoms by ureteral stent tube placement, but unfortunately, the cancerous lesion completely blocked the left ureteral opening. To protect the patient’s renal function and avoid further worsening of hydronephrosis, we opted for percutaneous left nephrostomy. After the patient’s lesion was controlled, the fistula was closed to improve the quality of life. (2) Considering impaired renal function, cisplatin was promptly replaced with carboplatin to reduce nephrotoxicity according to NCCN guidelines. (3) Regarding the data on the use of immune checkpoint inhibitors in cervical cancer, the timely addition of immune checkpoint inhibition and good results were achieved when limited efficacy of chemotherapy and severe myelosuppression were considered. KEYNOTE-158 (19) and CheckMate 358 (20) demonstrated that pembrolizumab and nivolumab are likely to prove useful against advanced and recurrent cervical cancer. A phase 3 clinical trial, KEYNOTE-826 (21), showed significantly improved OS and PFS in the pembrolizumab group in 617 patients with metastatic persistent recurrent cervical cancer not treated with chemotherapy, with a controlled safety profile. The main mechanism may be that the addition of chemotherapy to immunotherapy has a cumulative effect, and chemotherapy can disrupt the activity of immunosuppressive cells. Chemotherapy can also promote immune response by inducing apoptosis, upregulation of MHC1-like molecule expression and dendritic cell maturation in tumor cells. (4) Studies (22, 23) have shown that radiation therapy (RT) can induce immunomodulatory effects, alter the tumor micro-environment and upregulate the inflammatory cascade response. RT has been reported to increase PD-L1 expression and lead to immune failure, thus providing a rationale for the use of immunologic agents targeting PD-1/PD-L1 as a modality strategy in combination with RT. A meta-analysis showed comparable grade 3-4 toxicity in using ICI + RT compared to ICI alone in CNS melanoma metastases, NSCLC, and prostate cancer (24). (5) In patients who have endured chemotherapy for a considerable period, the lesions persist SD, and in the search for a more effective modality, anlotinib, in combination with sintilimab is chosen for maintenance therapy. Anlotinib is an oral multi-target anti-angiogenic TKI that inhibits vascular endothelial growth factor receptors (VEGFRs), fibroblast growth factor receptors, epidermal growth factor receptors, platelet-derived growth factor receptors and c-MET (25). Sintilimab, a fully human anti-PD1 IGG4-K monoclonal antibody, has been approved in China to treat classical Hodgkin’s lymphoma (26). Anlotinib can inhibit tumor angiogenesis and regulate tumor immune microenvironment, thereby enhancing the effect of PD-1 antibody (27, 28). Anlotinib has shown promising efficacy and safety in patients with advanced, recurrent cervical cancer (29). Simultaneously, combined immunotherapy has demonstrated promising efficacy in patients with advanced, recurrent cervical carcinoma (30). In this patient, the combination of the two drugs did achieve the effect of 1 + 1>2 during the application. (6) During the patient’s application of immune checkpoint inhibitors, the patient was closely monitored for the occurrence of adverse reactions, prompt drug control, and when the duration of the drug reached 1.5 years, the drug cycle was appropriately extended to slow discontinuation.

In this case, considering the characteristics of the patient and the changes in her condition, we conducted individualized treatment after obtaining her informed consent. After multiple changes in the treatment plan, the patient is currently in complete remission according to recent evaluations. In particular, in paclitaxel (albumin bound) combined with sintilimab, and in anlotinib combined with sintilimab, good efficacy was achieved, and no adverse reactions above grade 3 have been observed.

However, we also noted several problems in the whole case. (1) the financial constraints of the patient prevented the completion of genetic testing and PET-CT, which are completely self-funded in our country. (2) Steroids are an essential tool for treating immune-related side effects, but there is still controversy as to whether steroids affect the effect of immune checkpoint inhibitors. Previously we applied a chemotherapy regimen (docetaxel + carboplatin) that required dexamethasone pretreatment, for whether the efficacy was discounted, we are looking forward to proving it.

We will continue the current maintenance treatment plan and regularly follow up with the patient.

## Data availability statement

The original contributions presented in the study are included in the article/**Supplementary Material**. Further inquiries can be directed to the corresponding author.

## Ethics statement

Written informed consent was obtained from the individual(s) for the publication of any potentially identifiable images or data included in this article.

## Author contributions

YS is first authors, the major contributors in writing the manuscript. HC edited the manuscript and approved the final version. XM, LW, and XW were responsible for reviewing the literature and collecting the information of the patients. All authors listed have made a substantial, direct, and intellectual contribution to the work and approved it for publication.

## Funding

Liaoning Provincial Department of Education Fund Project: Project Number: ZF2019007.

## Conflict of interest

The authors declare that the research was conducted in the absence of any commercial or financial relationships that could be construed as a potential conflict of interest.

## Publisher’s note

All claims expressed in this article are solely those of the authors and do not necessarily represent those of their affiliated organizations, or those of the publisher, the editors and the reviewers. Any product that may be evaluated in this article, or claim that may be made by its manufacturer, is not guaranteed or endorsed by the publisher.
