# Supplementary figures and images for: Advanced primary vaginal squamous cell carcinoma: A case report and literature review
Source: Front Immunol. 2022 Nov 22;13:1007462. doi: 10.3389/fimmu.2022.1007462 (PMC9722770; doi:10.3389/fimmu.2022.1007462)

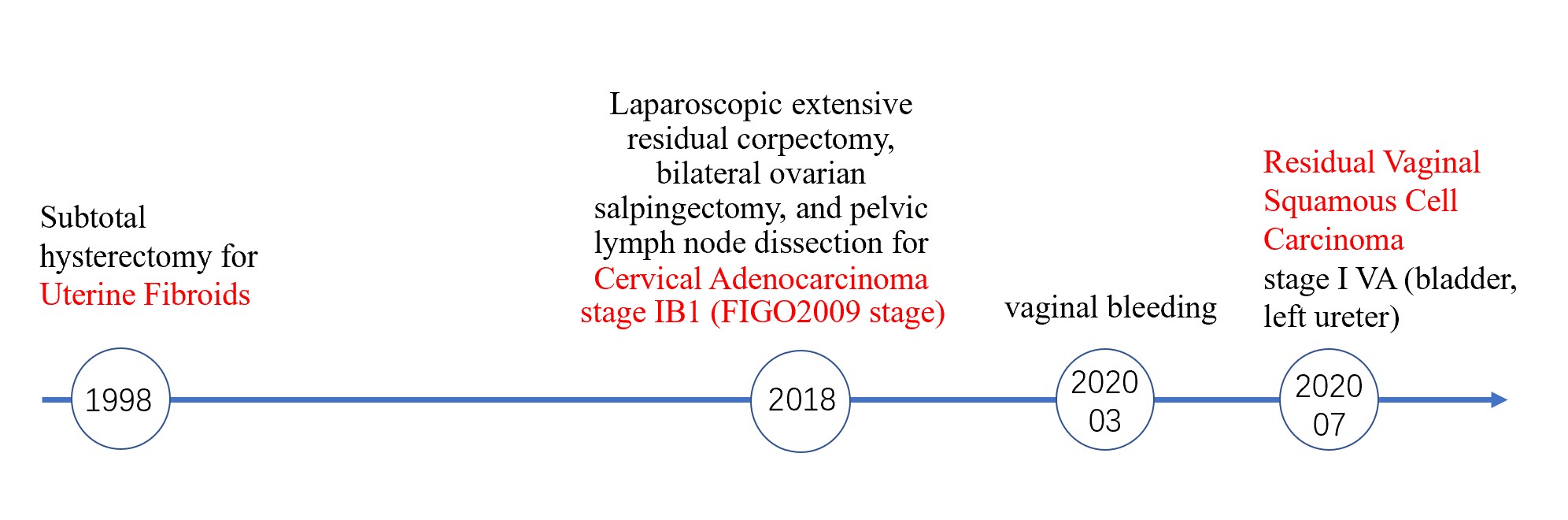

Supplement: Supplementary file 1 [file Image_1.jpg]

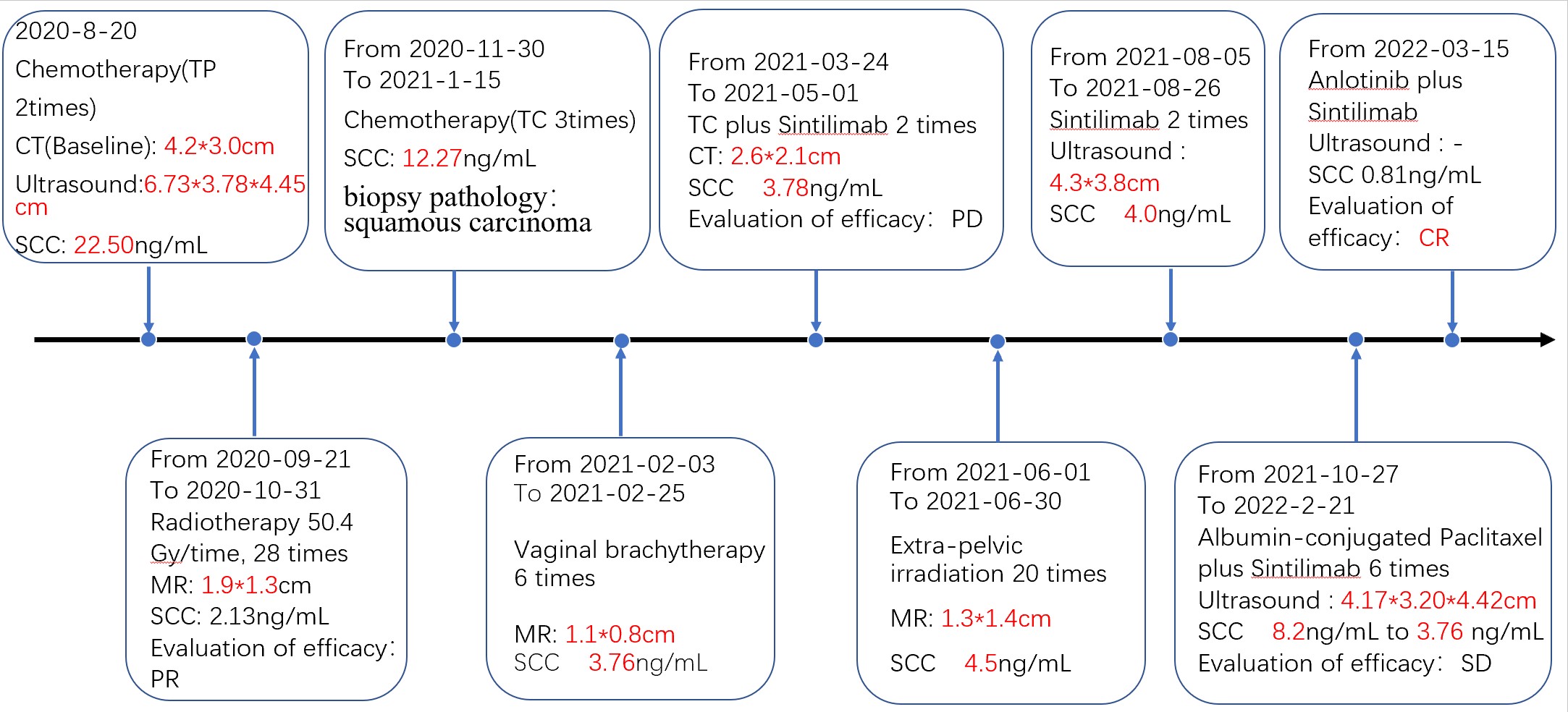

Supplement: Supplementary file 2 [file Image_2.jpg]
